# Supplementary material for: Evidence for topological contribution to spin shift current in antiferromagnetic Ti[image]C[image]
Source: Sci Rep. 2026 Jan 19;16:5753. doi: 10.1038/s41598-026-35948-x (PMC12894979; doi:10.1038/s41598-026-35948-x)
Supplement: Supplementary file 1 — Supplementary Information. [file 41598_2026_35948_MOESM1_ESM.pdf]

Supplementary Material for
"Evidence for topological contribution to spin shift current in antiferromagnetic Ti_4C_3"

I. EFFECT OF HUBBARD-U

The effect of varying the Hubbard-U parameter used and the magnetic configuration are summarized in Tab. (S1) and Tab. (S2).

TABLE S1: Effect of Hubbard U on the magnetic moment of outer Ti atoms in monolayer Ti_4C_3.

U (eV)	Magnetic Moment μ_B
0	0.64
1	0.68
2	0.73
3	0.78
4	0.81

TABLE S2: Total energies of monolayer Ti_4C_3 under different magnetic configurations as a function of the Hubbard U value.

U (eV)	NM (eV)	FM (eV)	AFM1 (eV)
0	-62.552	-62.675	-62.685
1	-59.446	-59.550	-59.619
2	-56.401	-56.442	-56.609
3	-53.413	-53.562	-53.659
4	-50.483	-50.650	-50.771

II. CRYSTAL STRUCTURE AND MAGNETIC ORDERING

The crystal structure and magnetic ordering for AFM1-AFM3 configurations is shown in Fig. (S1).

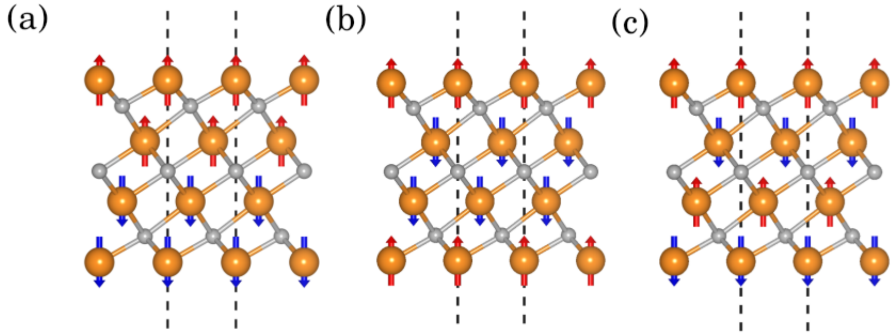

FIG. S1: Crystal structure of monolayer Ti_4C_3 with (a-c) AFM1−AFM3 magnetic ordering.

III. PHONON BAND STRUCTURE

We supply the phonon band structure of Ti_4C_3 in Fig. (S2).

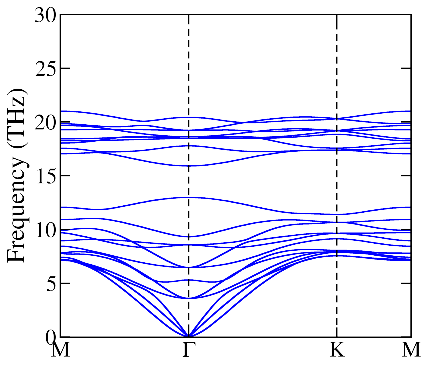

FIG. S2: Phonon band structure of monolayer Ti_4C_3.

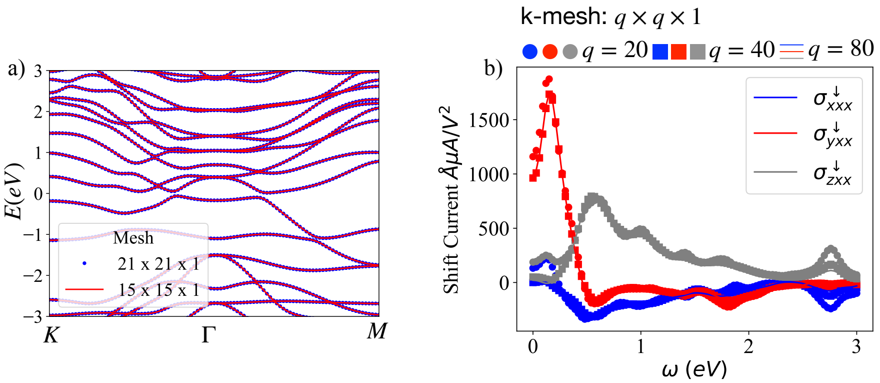

FIG. S3: (a) Band structure of Ti_4C_3 computed along high-symmetry path using Wannier90 tight binding model derived using a $15 \times 15 \times 1$ mesh of k-points and $21 \times 21 \times 1$ mesh. The band structure is overlapping demonstrating convergence. (b) Computation of spin-resolved shift current via the WannierBerri[1] software package. The computation is performed using three distinct choices of k-mesh, demonstrating convergence for a mesh of $80 \times 80 \times 1$ used in the main body.

IV. CONVERGENCE WITH RESPECT TO k-MESH

In this section we display the effect of utilizing two different choice of k-mesh on creation of the Wannier tight-binding model. This is shown in Fig. (S3)(a). We note that the two choices yield identical band structures demonstrating convergence of the model as a function of the mesh density. In the main body we utilize the denser mesh to perform computations.

We further study the effect of the k-mesh used in WannierBerri to compute the spin-resolved shift-current. In Fig. (S3)(b) we show the results of computing the shift-current for three choices of k-mesh. The results show convergence is reached for a $40 \times 40 \times 1$ mesh choice. In the main body we utilize a mesh of $80 \times 80 \times 1$ for all computations to ensure accuracy.

V. ENLARGED VIEW OF RTP INVARIANT

In Fig. 5 of the main body the Wannier center charge spectra for a subset of bands is analyzed and a quantized RTP invariant is demonstrated. In Fig. (S4) we enlarge the Wannier center charge spectra to demonstrate that the rapid evolution of the Wannier center charges for the spin-up sector near the Brillouin zone edge is not a computational artifact.

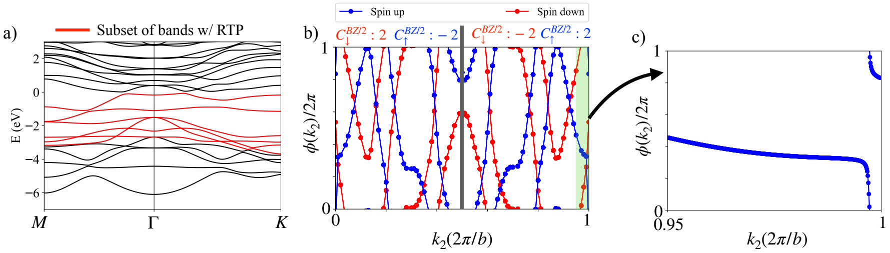

FIG. S4: (a) Band structure of Ti_4C_3 omitting spin-orbit coupling. (b) Results of Berry phase considering red bands in (a) as occupied. By decoupling the lower lying trivial bands a quantized RTP appears with a doubled invariant in both the spin-up and spin-down sectors. (c) Fine-grained computation of Berry phase for the spin-up bands in the region highlight in (b) near the zone edge at $k_2(2\pi/b) = 0$ (equivalent to $k_2(2\pi/b) = 1$), showing that the behavior of the Wannier center charges is not a computational artifact.

[1] S. S. Tsirkin, npj Comput Mater **7**, 33 (2021).
